# When Physicians Marry Physicians: Gender Inequities in Work Hours and Income

**DOI:** 10.1089/whr.2021.0048

**Published:** 2021-09-22

**Authors:** Xiaochu Hu, Michael Dill

**Affiliations:** Workforce Studies, Association of American Medical Colleges, Washington, District of Columbia, USA.

**Keywords:** work hours, gender inequities, dual-physician couples, same-occupation marriage, income

## Abstract

***Background:*** Physicians marry other physicians at a high rate, and theories suggest being married to a physician (MTP) may impact a physician's productivity in different ways. This impact may differ by gender and rurality of work location. This study empirically examines MTP's effects by gender and rurality of physicians' work location.

***Data and Method:*** This study uses both the Association of American Medical Colleges (AAMC) 2019 National Sample Survey of Physicians (*n* = 6,000) and the American Community Survey data 2006–2017 (*n* = 72,900). We conducted cross-sectional, multivariate analysis with interaction terms between MTP, gender, and rurality, controlling for various work and personal characteristics.

***Results:*** A female MTP physician works 2.9 fewer hours (95% confidence interval [CI]: −4.3 to −1.4, *p* = 0.000) per week than a female non-MTP physician, while a male MTP physician's weekly work hours are not significantly different from a male non-MTP physician's. Compared to non-MTP counterparts, male MTP physicians are more likely to have on-call work, and female MTP physicians are much less likely to have on-call work; male MTP physicians earn $6,635 more (95% CIs: $1,613–$11,657, *p* = 0.010) per year, while female MTP female physicians earn $5,018 less (95% CIs: −$10,684 to $648, *p* = 0.083). Furthermore, the MTP-associated gender differential effects are more prominent for physicians in rural areas than in urban areas. Results from both datasets are highly comparable.

***Conclusions:*** MTP's effects widen the gender gap in physicians' work hours, on-call probability, and earnings. Understanding and examining the mechanisms for these gender differential effects are essential to promote equity in the physician workforce.

## Introduction

Physicians marry other physicians at a high rate, and there are numerous reasons for and advantages of dual-physician marriage,^1,2^ as generally in same-occupation marriages.^3^ However, dual-physician marriages may not benefit each partner^†^ equally. Physicians typically work long hours and earn relatively high incomes, impacting their partners' labor supply and income in different ways.

First, the work hours of one partner may decrease as the work hours of the other increase.^4,5^ Research indicates that, in particular, it is the female in the partnership who decreases work hours to manage child or house care.^1,6^ This effect is often intensified with the presence of young children,^4,7^ or only salient with the presence of children.^8^ This relates to a large body of literature on “motherhood penalties” across all occupations, and indeed, motherhood penalties are stronger among highly skilled women.^9,10^ Second, in marriages where both partners earn a substantial salary, the subsequent family income may enable them to outsource housework to the market and work more hours at their jobs. For example, research found that female physicians in the United States spend less time in household activities that can be outsourced, such as housekeepers, gardeners, and chef services.^11^ Third, one partner's income may be substantial enough to enable the other partner to work fewer hours in the same way that wealth reduces labor supply in general.^12^ In the context of physicians married to physicians, how these different effects play out has seldom been empirically explored.

Practice location can further complicate the gender-differential impacts of being married to a physician (MTP). Studies have found that dual-college-educated couples, including physicians, are more likely to settle in urban areas, presumably because the more robust, diversified labor markets enable each partner to fulfill their career to a greater extent.^13–15^ Research on spousal mobility and earnings suggests that salary decreases due to mobility are often felt more by the females in dual college-educated marriages.^16^ However, studies have not yet determined how location rurality is associated with gender differences.

Our research adds to the literature in three important ways. First, it provides new, empirical evidence of MTP's association with physicians' work hours and income using two large-scale datasets and comprehensive statistical methods. Second, it examines the influence of geography on the MTP-associated gender gap. In addition to the knowledge this brings to physician workforce planning, this is also essential information for health care leaders striving to make their institutions more diverse, inclusive, and equitable. Third, in addition to work hours and income, recent literature points to “temporal flexibility” as the “last chapter” for closing the gender gap in labor market outcomes.^17^ By using on-call time as a distinct variable, we can measure possible MTP-associated gender disparities. Our findings on temporal flexibility provide pioneering evidence of a gender disparity in the physician workforce.

## Study Data and Methods

### Data

We applied the same statistical methods to two datasets: the Association of American Medical Colleges' (AAMC) National Sample Survey of Physicians (NSSP) and the American Community Survey (ACS).^18^ The NSSP sampled 6,000 physicians recruited online between February and March 2019. Sampling quotas were set across 24 age-sex-specialty strata based on power calculations (with *n* = 6,000, alpha = 0.05, for the two-sided alternative, for paired samples of data, the power is 1.000) and using the AMA's comprehensive database of physicians in the U.S. as a reference (requiring a minimum for each combination). The data were also weighted based on the AMA data to represent all practicing physicians in the United States regarding specialty group, gender, age group, and International Medical Graduate (IMG) status.^‡^ The NSSP was reviewed and approved by the AAMC Institutional Review Board. The ACS is an annual sample survey administered by the US Census Bureau and used in previous dual-physician family studies.^4,13,19^

Each dataset has its strength: the NSSP captures physician-specific information, such as medical specialty and whether physicians have spent any time on call during a typical week of work. The ACS has individual income information. Although broadly comparable, there are several critical variables that NSSP and ACS capture differently: MTP, weekly work hours, gender, and rurality. In the NSSP, MTP indicates whether a physician's partner is a trained physician and is currently working as one. In the ACS, MTP suggests that *both* partners report their current, main occupation as “physician.” Although NSSP's data on weekly work hours exclude on-call time, the ACS is not exclusive to physicians, and therefore, weekly work hours data do not indicate on-call time. Also, the ACS's gender variable is limited to “male” or “female.” The NSSP collects nonbinary gender information, so the “male” category in these analyses also includes “trans-male,” and the “female” category also includes “trans-female.” Finally, rurality in ACS is a four-category rurality indicator based on where physicians live: metropolitan area—in central/principal city (1), metropolitan area—central/principal city status indeterminable (mixed) (2), metropolitan area—not in central principal city (3), and rural areas (4). In NSSP, Rural-Urban Commuting Area (RUCA) codes are assigned based on a physician's primary workplace zip code. RUCA codes categorize rurality on a scale of 1 (most urban) to 10 (most rural).

Our analyses using NSSP and ACS yielded highly comparable results. This valuable comparison between the datasets offers more robust and validated conclusions. Supplementary Appendix SA1 lists basic statistics for all variables included in the study.

### Statistical methods

First, we conducted four ordinary least squares regressions—two using NSSP and two using ACS. The key explanatory variables were gender and MTP, a dummy variable indicating whether a physician is married to another physician. We also included an interaction term between gender and MTP, MTP×Gender, to allow MTP to affect each gender differently.

In analyses using NSSP data, the dependent variables were physician's weekly work hours and on-call status, a dummy variable indicating whether a physician had spent any time on call during a typical week of work. We controlled for the following covariates: age, age squared, marital status, partner's education level, working in a hospital, self-employed, years of postresidency practice, specialty group (medical specialties, primary care, surgery, and others), and IMG status. We cannot rule out the possibility that a physician is included together with their partner because we cannot link observations from the same family.

In analyses using ACS, the dependent variables were physician's weekly work hours and personal annual total income. We controlled for the following covariates: age, age squared, marital status, partner's education level, working in a hospital, self-employed, and foreign-born status. The regression model that used income as the dependent variable also controlled for weekly work hours. Because the ACS includes 10 years of pooled data, the regressions that used ACS data also had a year factor. We conducted all analyses at the individual level. No physician was included together with their partner to ensure that observations were independent of each other.

To better control for the effects of children on income and work hours, in all four models, we included two child-related covariates: a dummy variable indicating the presence of any child younger than 1 year and the total number of children younger than 5 years.

Next, we examined whether the effects of MTP by gender varied by levels of rurality of their practice location. We added rurality to the specification, as well as three two-way interactions (MTP×Gender, MTP×Rurality, and Gender×RURALITY) and one three-way interaction (MTP×Gender×RURALITY) in all models mentioned above.

We conducted all analyses using Stata 15.1.

## Main Findings

### Descriptive analysis using NSSP

Approximately 26% of all married physicians in the NSSP sample were married to other physicians, including 3% (*n* = 147) whose partners were not currently working as physicians. Among MTP physicians, females had a higher rate of not currently working as physicians than their male counterparts (65% vs. 35%, respectively). Among those who were not currently working as a physician, more females than males had not worked at all in the last year (51% vs. 43%, respectively). Among trained physicians who were working in a nonphysician capacity, 75% of males were working full time (defined as working 30 or more hours per week), compared to only 48% of females. In other words, female MTP physicians were more likely to reduce their labor supply, leave the physician workforce, or leave the workforce in general.

The NSSP data also showed that female physicians were much more likely to choose a practice location for their partners' careers. Twenty-two percent of all female physicians in the NSSP identified their partner's job as one of the main reasons for location choice, compared to only 10% of male physicians. In addition, among MTP physicians, 24% of female physicians indicated that their partner's job was one of the main reasons for choosing their first practice location, compared to 16% of males.

### Work hours, on-call probability, and income

**FIG. 1.**
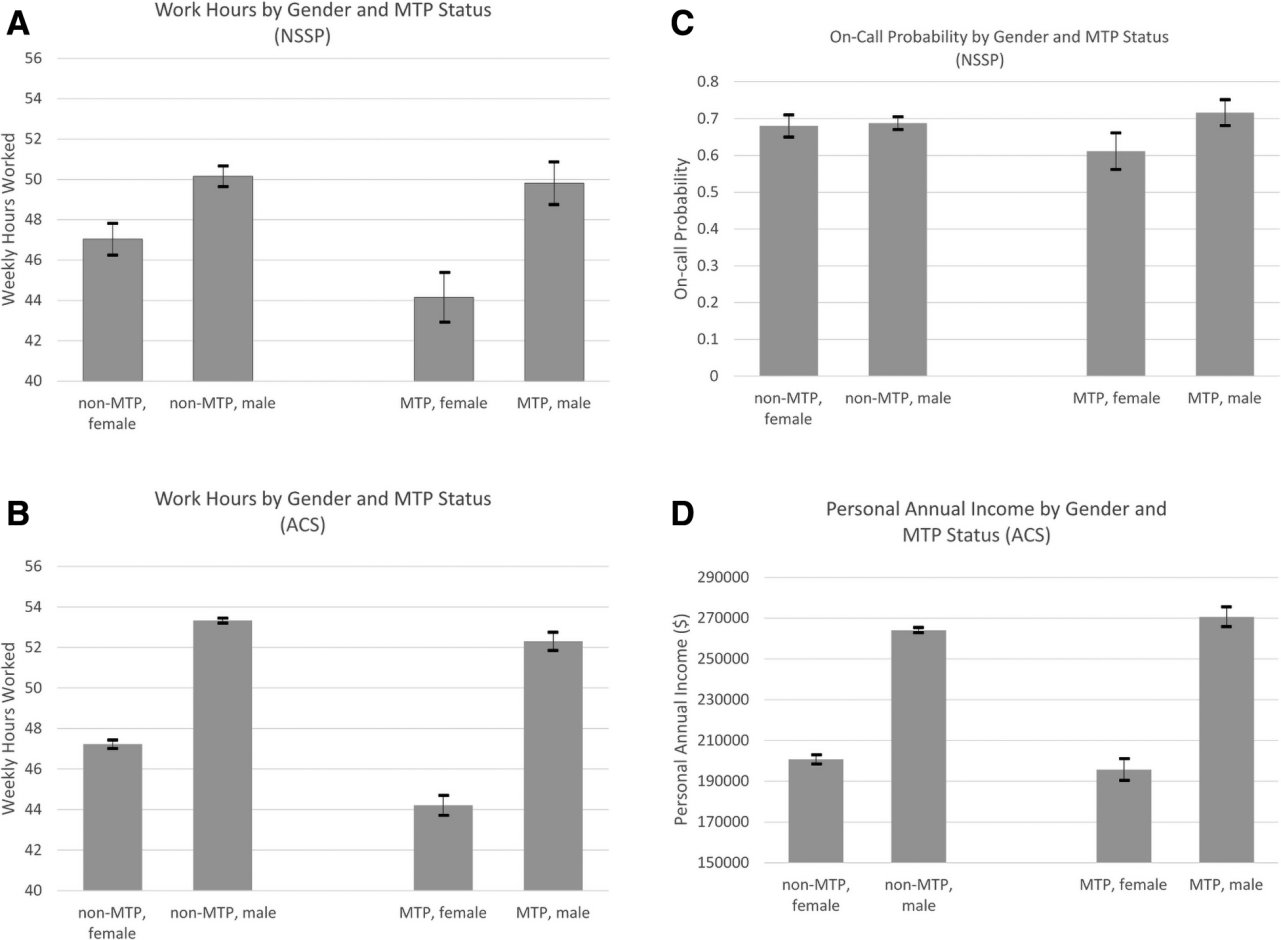
Weekly work hours, on-call probability, and annual personal income with 95% CIs, by gender and being MTP status. Source: authors' analyses of data from the 2019 NSSP **(A and C**; *n* = 4,683**)**, and 2006–2017 ACS 1-year estimates **(B and D**; *n* = 72,900**)**. Samples of NSSP are limited to full-time working physicians (all NSSP physicians are not residents). Samples of ACS limited to full-time working, nonresident physicians, and physicians reporting positive annual income. Residents in ACS are defined as physicians younger than 35 years, working in a hospital, and with an annual wage income <$80,000. Notes: the gender differential MTP effects (the interaction terms between gender and MTP status in multiple regressions) are all statistically significant **(***p* < 0.01 for **A, C, and D**; *p* < 0.001 for **B)**. No physician in the same household is included in the regressions. All analyses were weighted. See Supplementary Appendix SA2 for additional information. ACS, American Community Survey; CI, confidence interval; MTP, married to a physician; NSSP, National Sample Survey of Physicians.

Figure 1A–D illustrates the predicted margins of outcome with 95% confidence intervals (CIs) by gender and MTP status, controlling for other covariates (see Supplementary Appendix SA2 for full regression results). MTP physicians reported working fewer hours per week than their non-MTP counterparts. Still, the within-gender MTP and non-MTP differential were much larger for female than male physicians (Fig. 1A). A female MTP physician worked 2.9 fewer hours (95% CI: −4.3 to −1.4, *p* = 0.000) than a female non-MTP physician. A male MTP physician worked only 0.3 fewer hours (95% CI: −1.5 to 0.8, *p* = 0.567) than a male non-MTP physician, and this difference is not statistically significant. From the between-gender perspective, non-MTP physicians have a gender difference of 3.1 hours a week (male more than female, 95% CIs: 2.2–4.1, *p* = 0.000). For MTP physicians, this gender gap is 5.7 hours (male more than female, 95% CIs: 4.1–7.2, *p* = 0.000).

Analyses using ACS data (Fig. 1B) reached a similar conclusion, although there were larger between-gender gaps for both non-MTP (6.1 hours) and MTP physicians (8.1 hours). As previously mentioned, ACS data did not provide the ability to control for physicians' medical specialty or to separate on-call time from typical work hours, which we believe explains the larger gender gap.

Figure 1C shows that a male MTP physician had a 7% higher chance of having any on-call days than a male non-MTP physician. Conversely, female MTP physicians had a 28% *lower* chance of having on-call days than female non-MTP physicians. Thus, from the between-gender perspective, the gender gap in on-call probability between *non*-MTP physicians was much smaller than between MTP males and MTP females (5% vs. 41% points, respectively).

Last, MTP was associated with $6,635 increase in annual income (95% CIs: $1,613–$11,657, *p* = 0.010) for male physicians, but $5,018 *decrease* (95% CIs: −$10,684 to $648, *p* = 0.083) for female physicians after controlling for work hours (Fig. 1D). When we analyzed any between-gender difference, the gender gap was smaller for non-MTP than MTP physicians ($63,300 vs. $75,000).

### Rurality

**FIG. 2.**
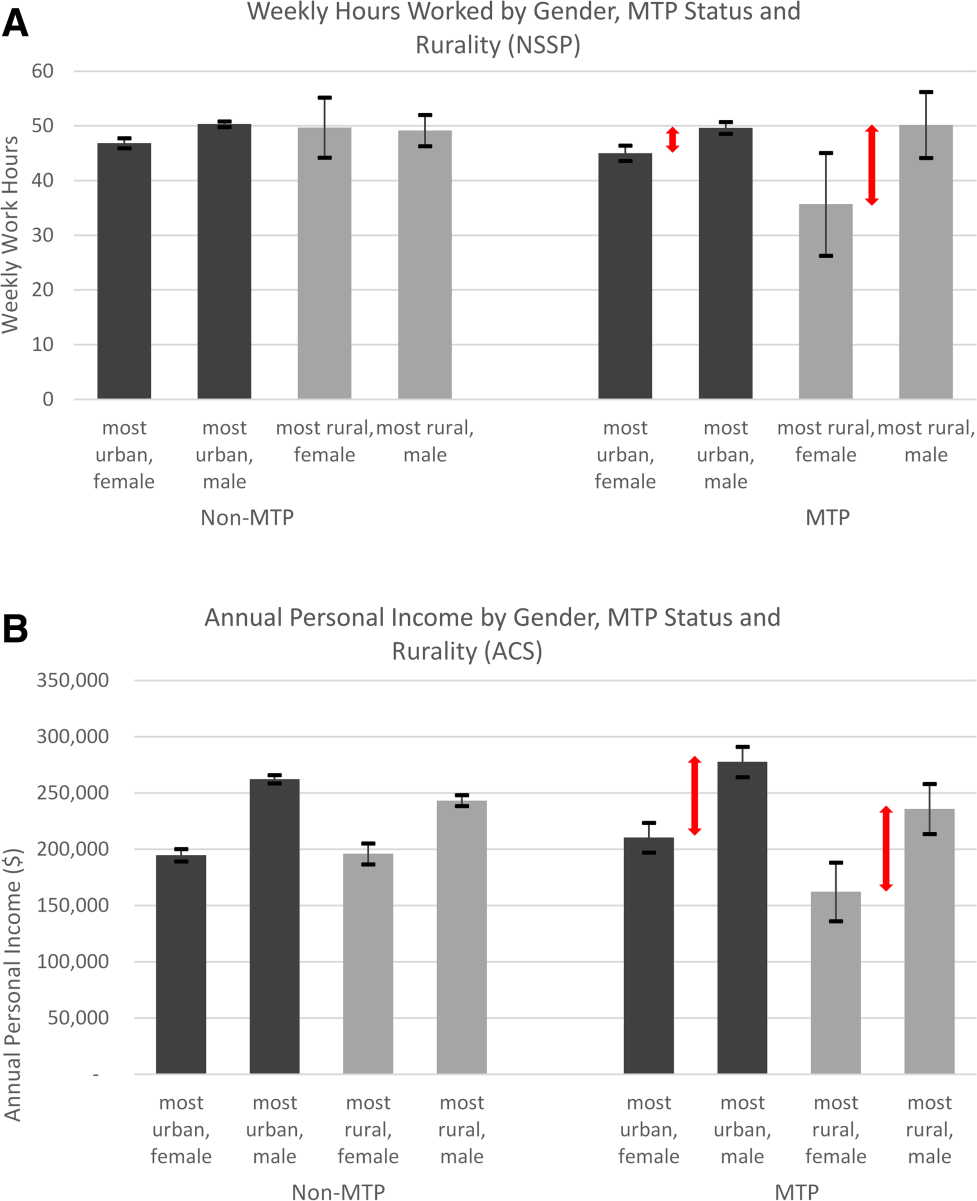
Weekly work hours and annual personal income with 95% CIs, by location rurality, gender, and MTP status. Source: authors' analyses of data from the 2019 NSSP and 2006–2017 ACS 1-year estimates. Samples of NSSP are limited to full-time working physicians (all NSSP physicians are not residents). Samples of ACS limited to full-time working, nonresident physicians, and physicians reporting positive annual income. Residents in ACS are defined as physicians younger than 35 years, working in a hospital, and with an annual wage income less than $80,000. Notes: regression triple interaction terms are statistically significant for both graphs **(A**: *p* < 0.05; **B**: *p* < 0.05**)**; the “most urban area” is defined as “metropolitan area —in central/principal city” (1) in ACS and RUCA code of 1 in NSSP. The “most rural area” is defined as “rural areas” (4) in ACS and RUCA code of 10 in NSSP. All analyses weighted. See Supplementary Appendix SA3 for additional information. RUCA, Rural-Urban Commuting Area.

Next, we examined whether the gender-differentiated effects of MTP differed by rurality of practice location. The triple interaction terms were statistically significant in the NSSP work hours and ACS income regressions and not significant in the NSSP on-call probability and ACS work hour regressions (see Supplementary Appendix SA3 for full regression results). Figure 2A and B illustrate predicted margins of outcome with 95% CIs of the two models with significant triple interactions by gender, MTP status, and rurality, controlling for other covariates (for the sake of simplicity, only the lowest and highest rurality scores are plotted).

Figure 2A shows that in the most rural areas (rurality score = 10), MTP male physicians work 14.5 hours more per week than MTP females. However, this difference was only 4.6 hours in the most urban areas (rurality score = 1). Similarly, the gender discrepancy in predicted personal annual income for MTP physicians was greater in rural areas than in urban areas (Fig. 2B). For example, in the most urban areas, MTP males earned $65,170 more than MTP females. In the most rural areas, however, this difference increased to $90,770. In sum, MTP status in rural areas was associated with a larger gender discrepancy in work hours and income than in urban areas. These findings support and enrich previous research demonstrating that dual-physician couples who live in rural areas have more divergent work hours, and the females are more negatively impacted.^14,15^

## Discussion

Our descriptive analyses using NSSP data suggest that female MTP physicians are more likely to leave the physician workforce than MTP male physicians. Female MTP physicians are also more likely than male MTP physicians to drop out of the labor market or work only part time. Our regression analyses indicate that MTP status is associated with reduced weekly work hours for female physicians, but barely for male physicians. The dual-physician income that MTP status brings only reduces the labor supply for female physicians, echoing the theories that wealth reduces labor supply and that females are more likely to experience a negative impacts than males.^20,21^ We believe two main reasons contribute to this gender-differential MTP impact on physicians' work hours.

First, uneven gender distribution across medical specialties may drive this discrepancy. Although women have comprised a near majority of all medical school graduates since 2007–2008,^22^ their representation within individual specialties varies tremendously, and the women-dominated specialties tend to earn less.^23,24^ Dual-physician couples who face the challenge of balancing work and household responsibilities may choose to have the lower-paid partner, which may disproportionately be the females, reduce their work hours. Carr et al. demonstrated that female physicians who have reduced their work hours were more likely to be generalists, a lower-paid specialty.^25^ Although having the lower-paid partner reduce work hours can be an economically efficient choice for families, it can substantially undermine female participation in the physician workforce and reinforce gender inequities in both hours worked and individual income.

Second, females in dual-physician marriages may be the ones to reduce their hours or leave the workforce regardless of income because of inequitable social norms. Traditionally, females bear the brunt of domestic responsibilities. For example, Lyu's 2019 research found that some female surgeons, a highly paid specialty, left the workforce due to the difficulty of balancing a procedural career with domestic tasks.^26^

We also found that MTP status is associated with an enlarged gender gap in on-call probability. In addition, because our income analyses controlled for work hours, when and how physicians work may impact income more than their actual amount of hours. These findings indicate that physicians with tasks demanding more “temporal flexibility”—the ability to work hours when and as needed—may be able to earn more than physicians whose domestic responsibilities require stricter schedules. Physicians with greater temporal flexibility may therefore be more likely to advance in their careers rapidly. Further research on physician on-call time, income, and gender difference is needed.

Furthermore, MTP's gender-diverging effects on work hours and income were higher in rural areas than in urban ones. One potential explanation for this could be geographical differences in job markets. Previous research suggests that metropolitan areas may be more attractive to dual-educated, highly skilled couples because the larger and robust job markets afford more opportunities for each partner to find meaningful, relevant employment.^13,15,16,27,28^ It follows, then, that smaller labor markets in rural areas limit the ability of each marriage partner to find a job, regardless of their skill or education level. In addition, as couples are more likely to relocate due to the male's employment, female partners may disproportionately find suboptimal work in rural areas or no work at all. By proactively focusing on career opportunities for both partners, recruiting firms and physician employers may more successfully attract physician couples to mitigate rural areas' recruitment and retention difficulties.

These findings assume additional weight during the current COVID-19 pandemic. The COVID-related increase in gender inequality has already been observed in various research fields and academia, including the practice of medicine.^29–32^ Given widespread school and child care closures and the nature of their work, dual-physician couples face a dire challenge. We worry that female MTP physicians are more likely to reduce their work hours or exit the labor force entirely.

This research has limitations. First, we recognize NSSP's sampling method is not entirely random and may have potential sample bias. However, by supplementing NSSP with ACS, we are confident we can generalize our findings. Second, our analyses do not suggest a causal relationship between MTP and work hours, on-call probability, or income. Future studies using longitudinal data measuring MTP status and labor market outcomes at multiple points in time would shed light on whether physicians are more likely to change work hours because they marry another physician.

Finally, although our datasets included a small number of same-sex couples, our analyses did not differentiate whether MTP is hetero- or same-sex because there are not enough data to support analysis with same-sex MTP as a separate category.

## Conclusion and Policy Implications

Our study uses nationally representative physician survey data that reflect numerous specialties and provide valuable information not available in other datasets. By supplementing the NSSP with the ACS, this research offers new evidence for both the same-occupation marriage and physician gender inequities literature. Our analysis of on-call work also highlights the importance of “temporal flexibility”—a dimension of work effort beyond simply the amount of work hours. Furthermore, this research emphasizes the impact of rurality on MTP physicians, informing efforts to recruit physicians to rural areas. The patterns of work and income associated with MTP status can perpetuate gender inequity in the US physician workforce and other high-skilled occupations, as well.

It is important to note that we controlled for parenthood in our analyses, and therefore any association between MTP and outcomes is confirmed regardless of observations' parental status. Thus, policies only addressing childcare needs, while important, will not sufficiently mitigate MTP's gender differential effects. Instead, policies and programs must focus on equitable gender representation across all specialties. For example, promoting all specialty choices to medical students and proactively broadening female leadership may more effectively alleviate the gender concentration in specialties and pay gap and address the root cause of any MTP-associated gender disparity. More robust employment opportunities in rural areas may also mitigate these effects and direct the physician workforce toward gender equity and inclusion.

## Supplementary Material

Supplemental data

Supplemental data

Supplemental data

## Supplementary Material

Supplementary Appendix SA1

Supplementary Appendix SA2

Supplementary Appendix SA3

## Abbreviations Used

AAMC: Association of American Medical Colleges
ACS: American Community Survey
CI: confidence interval
IMG: International Medical Graduate
MTP: married to a physician
NSSP: National Sample Survey of Physicians
RUCA: Rural-Urban Commuting Area

## Notes

^†^ Our study defines marriage broadly, including committed unmarried relationships. To support this inclusion, we have opted to use the term “partners” rather than “spouses.”

^‡^ More details about NSSP can be found at https://www.aamc.org/media/44246/download
