# Supplementary material for: When Physicians Marry Physicians: Gender Inequities in Work Hours and Income
Source: Womens Health Rep (New Rochelle). 2021 Sep 22;2(1):422–9. doi: 10.1089/whr.2021.0048 (PMC8524735; doi:10.1089/whr.2021.0048)
Supplement: Supplemental data [file Supp_AppTableS1.docx]

**Appendix Tables**

**Appendix A. Basic statistics for key variables**

|  | **mean** | **St dev** | **max** | **min** | **Obs N** |
| --- | --- | --- | --- | --- | --- |
| **NSSP** |  |  |  |  |  |
| MTP (of all married physicians) | 0.22 | 0.42 | 1 | 0 | 4,766 |
| Weekly Work hour (excluding call) | 49.17 | 13.58 | 150 | 21 | 5,627 |
| On-call probability | 0.66 | 0.48 | 1 | 0 | 5,620 |
| Male | 0.65 | 0.48 | 1 | 0 | 5,639 |
| Rurality (RUCA code, 1-10) | 1.42 | 1.40 | 10 (most rural) | 1 (most urban) | 5,531 |
| **ACS** |  |  |  |  |  |
| MTP* (of all physicians) | .07 | .25 | 1 | 0 | 88,991 |
| Weekly Work hour | 51.45 | 14.11 | 99 | 21 | 88,991 |
| Annual personal Income | 236,452 | 153,946 | 1,343,000 | 1 | 88.991 |
| male | .69 | .46 | 1 | 0 | 88,991 |
| Rurality (1-4) | 2.308 | .83 | 4 (most rural) | 1 (most urban) | 83,211 |
| Married | .77 | 0.42 | 1 | 0 | 88,991 |

Note: *In ACS, for dual-physician household, we randomly chose one physician to include in the regression analysis to avoid inter-dependency. This is the reason that the variable MTP mean is smaller than the actual percent of physicians being married to physicians.
