# Supplementary material for: When Physicians Marry Physicians: Gender Inequities in Work Hours and Income
Source: Womens Health Rep (New Rochelle). 2021 Sep 22;2(1):422–9. doi: 10.1089/whr.2021.0048 (PMC8524735; doi:10.1089/whr.2021.0048)
Supplement: Supplemental data [file Supp_AppTableS2.docx]

**Appendix B. Full regression estimators DD (interaction)**

| data | NSSP | | ACS | |
| --- | --- | --- | --- | --- |
| Model | (A) | (C) | (B) | (D) |
|  | Weekly work hours | On-call probability | Weekly work hours | Personal annual income (dollar) |
| Male | 3.123*** | 0.0374 | 6.101*** | 63311.9*** |
|  | (6.44) | (0.43) | (48.90) | (46.85) |
| MTP | -2.881*** | -0.317* | -3.027*** | -5017.5 |
|  | (-3.88) | (-2.35) | (-11.17) | (-1.74) |
| Interaction - Male and MTP | 2.532** | 0.462** | 2.000*** | 11652.2** |
|  | (2.72) | (2.79) | (5.55) | (3.03) |
| Age (years) | 0.927*** | 0.184*** | 0.587*** | 17695.7*** |
|  | (5.63) | (6.60) | (16.21) | (45.79) |
| Age squared | -0.00688*** | -0.00155*** | -0.00786*** | -157.7*** |
|  | (-4.58) | (-6.12) | (-22.94) | (-43.03) |
| IMG Status / Foreign-born | -0.164 | -0.113 | -0.378*** | -9534.3*** |
|  | (-0.37) | (-1.33) | (-3.33) | (-7.88) |
| Having high skilled partner | 0.103 | -0.0729 | 0.198*** | 2897.9*** |
|  | (0.24) | (-0.96) | (4.88) | (6.69) |
| Work experience (years) | -0.363*** | -0.0252** |  |  |
|  | (-7.46) | (-2.84) |  |  |
| Total children under 5 years old | -1.166* | 0.129 | -0.799*** | 10589.0*** |
|  | (-2.39) | (1.53) | (-7.67) | (9.52) |
| With infant | -1.017 | -0.407* | 0.697** | -15393.9*** |
|  | (-0.93) | (-2.24) | (2.63) | (-5.45) |
| 1.Medical specialty (default) | 0 | 0 |  |  |
|  | (.) | (.) |  |  |
| 2.Other Specialty | -1.422* | -0.848*** |  |  |
|  | (-2.48) | (-8.52) |  |  |
| 3.Primary care | -1.527** | -0.167 |  |  |
|  | (-2.71) | (-1.81) |  |  |
| 4.surgery | 1.824** | 0.271* |  |  |
|  | (2.92) | (2.41) |  |  |
| hospital | 3.659*** | 0.304*** | 1.576*** | 7645.1*** |
|  | (7.55) | (3.58) | (14.04) | (6.38) |
| Self-employed | 0.512 | 0.374*** | 0.999*** | 11225.0*** |
|  | (1.18) | (4.97) | (8.16) | (8.60) |
| Married |  |  | 0.146 | 20327.7*** |
|  |  |  | (0.53) | (6.94) |
| Weekly hours worked |  |  |  | 1506.1*** |
|  |  |  |  | (38.15) |
| 2006.year dummy |  |  | 0 | 0 |
|  |  |  | (.) | (.) |
| 2007.year dummy |  |  | 0.313 | 13229.2*** |
|  |  |  | (1.24) | (4.93) |
| 2008.year dummy |  |  | -0.202 | 21195.7*** |
|  |  |  | (-0.81) | (7.95) |
| 2009.year dummy |  |  | 0.0372 | 23270.2*** |
|  |  |  | (0.15) | (8.68) |
| 2010.year dummy |  |  | -0.0587 | 22574.6*** |
|  |  |  | (-0.23) | (8.45) |
| 2011.year dummy |  |  | -0.0719 | 22958.4*** |
|  |  |  | (-0.29) | (8.68) |
| 2012.year dummy |  |  | -0.749** | 34154.6*** |
|  |  |  | (-3.03) | (12.95) |
| 2013.year dummy |  |  | -0.0977 | 48367.6*** |
|  |  |  | (-0.40) | (18.41) |
| 2014.year dummy |  |  | -0.482 | 56173.5*** |
|  |  |  | (-1.93) | (21.05) |
| 2015.year dummy |  |  | -0.530* | 67741.2*** |
|  |  |  | (-2.15) | (25.73) |
| 2016.year dummy |  |  | -0.569* | 79741.3*** |
|  |  |  | (-2.30) | (30.26) |
| 2017.year dummy |  |  | -0.387 | 87193.9*** |
|  |  |  | (-1.58) | (33.39) |
| Constant | 25.43*** | -3.742*** | 35.95*** | -441975.3*** |
|  | (5.78) | (-5.00) | (34.45) | (-39.43) |
| N | 4683 | 4678 | 72916 | 72897 |
| R-2 | 0.0696 | 0.0420 | 0.0680 | 0.1374 |

Note: * p<0.05, ** p<0.01, *** p<0.001. NSSP data restricted to full time practicing physicians, t statistics in parentheses
