# Supplementary material for: When Physicians Marry Physicians: Gender Inequities in Work Hours and Income
Source: Womens Health Rep (New Rochelle). 2021 Sep 22;2(1):422–9. doi: 10.1089/whr.2021.0048 (PMC8524735; doi:10.1089/whr.2021.0048)
Supplement: Supplemental data [file Supp_AppTableS3.docx]

**Appendix C. Full regression estimators with DDD (triple interactions)**

| Data | NSSP | ACS | NSSP | ACS |
| --- | --- | --- | --- | --- |
| Model | (A) | (B) | (3) (C) | (4) (D) |
|  | Weekly work hour | Personal annual income | On-call probability | Weekly work hour |
| Male | 3.947*** | 67729.4*** | 0.0515 | 5.071*** |
|  | (5.45) | (16.85) | (0.42) | (10.70) |
| MTP | -0.478 | 19363.1* | -0.335* | -3.464*** |
|  | (-0.41) | (2.13) | (-1.97) | (-3.34) |
| Rurality | 0.319 | 824.5 | -0.00782 | -0.469** |
|  | (0.99) | (0.59) | (-0.15) | (-2.89) |
| Interaction – Male × rurality | -0.450 | -1912.7 | 0.0156 | 0.476* |
|  | (-1.24) | (-1.18) | (0.27) | (2.52) |
| Interaction -rurality × MTP | -1.356* | -10478.8** | 0.0421 | 0.203 |
|  | (-2.14) | (-2.81) | (0.54) | (0.48) |
| Interaction -male × MTP | -0.407 | -11090.8 | 0.156 | 2.629 |
|  | (-0.29) | (-0.90) | (0.38) | (1.90) |
| Interaction -male × MTP × rurality | 1.546* | 10446.6* | 0.194 | -0.264 |
|  | (2.08) | (2.08) | (0.47) | (-0.46) |
| Age | 1.006*** | 17869.6*** | 0.178*** | 0.589*** |
|  | (6.00) | (44.62) | (6.28) | (13.05) |
| Age squared | -0.00748*** | -158.9*** | -0.00148*** | -0.00790*** |
|  | (-4.90) | (-41.82) | (-5.76) | (-18.96) |
| Married | 0 | 20350.5*** | 0 | 0.167 |
|  | (.) | (6.71) | (.) | (0.47) |
| IMG status / Foreign born | -0.332 | -10292.6*** | -0.118 | -0.480*** |
|  | (-0.67) | (-8.26) | (-1.36) | (-3.31) |
| High skilled spouse | 0.275 | 2882.6*** | -0.0409 | 0.211*** |
|  | (0.63) | (6.36) | (-0.52) | (4.02) |
| Working experience (years) | -0.386*** |  | -0.0258** |  |
|  | (-7.61) |  | (-2.87) |  |
| Total children under 5 years old | -1.123* | 10697.0*** | 0.147 | -0.825*** |
|  | (-2.33) | (9.32) | (1.72) | (-6.03) |
| With infant | -1.078 | -15077.3*** | -0.483** | 0.605 |
|  | (-0.99) | (-5.19) | (-2.61) | (1.53) |
| 1.SPECIALTY GROUP: Medical specialties (default) | 0 |  | 0 |  |
|  | (.) |  | (.) |  |
| 2.SPECIALTY_GROUP: others | -1.600** |  | -0.875*** |  |
|  | (-2.73) |  | (-8.68) |  |
| 3.SPECIALTY_GROUP: primary care | -1.411** |  | -0.160 |  |
|  | (-2.67) |  | (-1.70) |  |
| 4.SPECIALTY_GROUP: surgery | 1.964** |  | 0.262* |  |
|  | (3.26) |  | (2.30) |  |
| Working in hospital | 3.321*** | 7267.8*** | 0.269** | 1.594*** |
|  | (6.74) | (5.85) | (3.13) | (11.31) |
| Self-employed | 0.176 | 11327.9*** | 0.347*** | 1.029*** |
|  | (0.41) | (8.34) | (4.56) | (6.83) |
| 2006.year effect |  | 0 |  | 0 |
|  |  | (.) |  | (.) |
| 2007.year effect |  | 13248.7*** |  | 0.298 |
|  |  | (4.80) |  | (0.98) |
| 2008.year effect |  | 21434.7*** |  | -0.279 |
|  |  | (7.81) |  | (-0.95) |
| 2009.year effect |  | 22871.3*** |  | 0.0187 |
|  |  | (8.29) |  | (0.06) |
| 2010.year effect |  | 21735.9*** |  | -0.155 |
|  |  | (7.90) |  | (-0.51) |
| 2011.year effect |  | 23064.8*** |  | -0.124 |
|  |  | (8.46) |  | (-0.39) |
| 2012.year effect |  | 33588.1*** |  | -0.860** |
|  |  | (12.28) |  | (-2.80) |
| 2013.year effect |  | 49752.7*** |  | -0.186 |
|  |  | (18.24) |  | (-0.57) |
| 2014.year effect |  | 55740.0*** |  | -0.481 |
|  |  | (20.16) |  | (-1.56) |
| 2015.year effect |  | 68556.2*** |  | -0.675* |
|  |  | (25.04) |  | (-2.22) |
| 2016.year effect |  | 78845.0*** |  | -0.706* |
|  |  | (28.82) |  | (-2.33) |
| 2017.year effect |  | 86499.8*** |  | -0.437 |
|  |  | (31.99) |  | (-1.45) |
| Weekly work hour |  | 1499.8*** |  |  |
|  |  | (36.57) |  |  |
| Constant | 23.23*** | -447888.8*** | -3.570*** | 36.90*** |
|  | (5.15) | (-37.41) | (-4.68) | (26.48) |
| N | 4597 | 68075 | 4593 | 68075 |

Note: * p<0.05, ** p<0.01, *** p<0.001. NSSP data restricted to full time practicing physicians, t statistics in parentheses
